# Supplementary material for: Synthetic Peptide Purification via Solid-Phase Extraction with Gradient Elution: A Simple, Economical, Fast, and Efficient Methodology
Source: Molecules. 2019 Mar 28;24(7):1215. doi: 10.3390/molecules24071215 (PMC6479624; doi:10.3390/molecules24071215)
Supplement: Supplementary file 1 [file molecules-24-01215-s001.pdf]

molecules

MDPI

Supplementary Materials

Synthetic Peptide Purification via Solid-Phase Extraction with Gradient Elution: A Simple, Economical, Fast, and Efficient Methodology

Diego Sebastián Insuasty Cepeda [1], Héctor Manuel Pineda Castañeda [1], Andrea Verónica Rodríguez Mayor [1], Javier Eduardo García Castañeda [2], Mauricio Maldonado Villamil [1], Ricardo Fierro Medina [1] and Zuly Jenny Rivera Monroy [1,*]

* Correspondence: zjriveram@unal.edu.co; Tel.: +57-1-3165000 (ext. 14436)

Herein an example of peptide purification via RP-SPE and gradient elution is shown

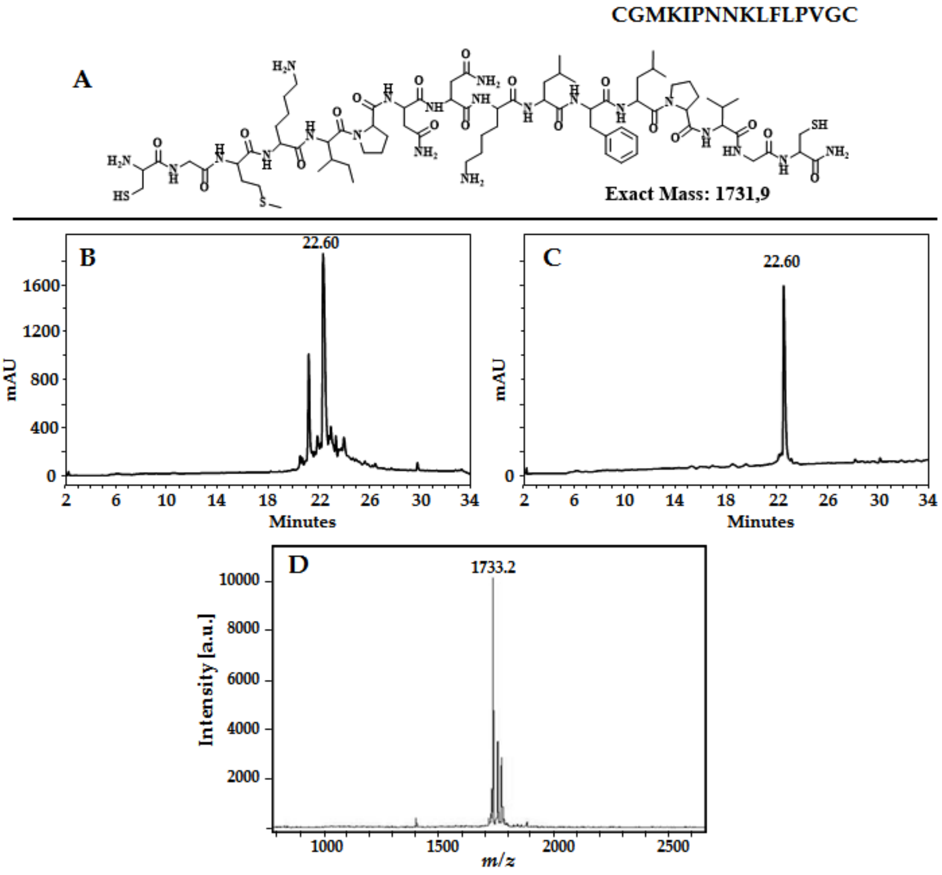

Figure S1. Purification of a peptide containing seventeen amino acids. Sequence and structure (**A**), chromatographic profile of crude (**B**) and purified (**C**) product. For signal at t_R: 22.60 min, the percentage of peak area is 45% (B) and 85% (C). Calculated $\%B_e$, for target peak, was 21.6% B, the purest fraction contained 23% B. Purification yield was 13%. MALDI-TOF spectrum of purified peptide (**D**); it is observed a signal at *m*/*z* 1733.2 corresponding to the $[M + H]^+$ specie. Fractions collected were analyzed by RP-HPLC in an Agilent 1200 liquid chromatograph with UV-Vis detector (210 nm). An Eclipse XDB-C18 column (3.5 µm; 4.6 × 150 mm, t_0 = 2.2 min) was used, using elution gradient of 5/5/50/100/100/5/5% solvent B (TFA 0.05% in ACN) in 0/2/47/49/55/55.1/70 min, a flow rate of 1.0 mL/min, t_D 1.8 min. 10 µL of sample solution (1 mg/mL) were injected.
